# Combined laparoscopic cholecystectomy and incisional hernia repair: a proposal for standardised technique

**DOI:** 10.1308/003588412X13373405387096i

**Published:** 2012-05

**Authors:** N Vettoretto, M Bartoli, G Montori, M Giovanetti

**Affiliations:** ^1^Mellino Mellini Hospital, Chiari,Italy; ^2^University of Brescia,Italy

## BACKGROUND

The concomitant presence of symptomatic cholelithiasis and a large median ventral hernia invites a simultaneous laparoscopic approach, previously described for small defects.^1,2^ We suggest a standardised method to treat these conditions simultaneously.

## TECHNIQUE

**Figure 1:**
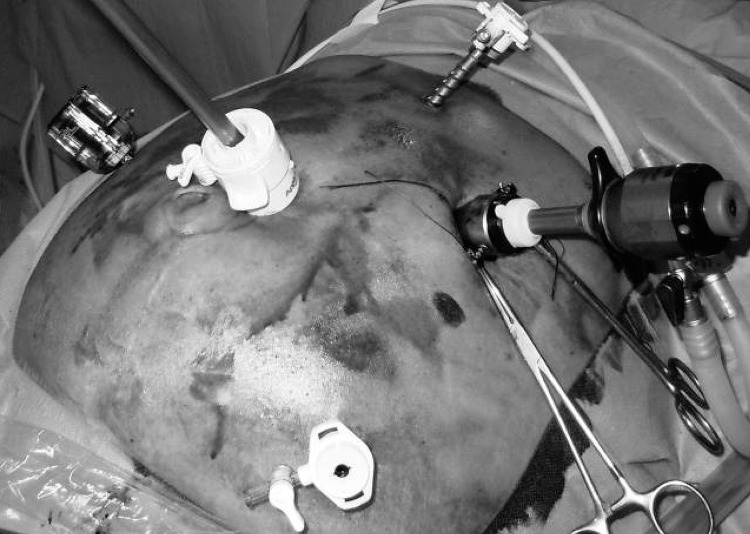
Trocar position

An open Hasson entry in the left hypochondrium, at least 5cm lateral to the defect edge, and two 5mm trocars in the subxiphoid area and left iliac fossa serve to take down hernia sac adhesions and completely expose the abdominal wall defect. Another 10mm trocar is inserted in the midline just cephalad (or caudal) to the defect to receive the camera, together with a 5mm access in the right flank (Fig 1). Cholecystectomy is then performed in the standard French fashion, taking special care to avoid accidental perforation.The gallbladder is retrieved in a bag, through the open entry,to prevent accidental spillage. A double-layer of intraperitoneal mesh is placed to cover the defect and the midline trocar site with an adequate overlap and fixation.

## DISCUSSION

We have standardised this technique through use in four patients,without morbidity, with a brief operative time (65 minutes) and a short hospital stay (2 days). The use of five trocars allows the same confidence with the view as in two separate procedures (in order to prevent biliary injuries) and permits the defect repair with an adequate inlay mesh while minimising the risks of contamination and recurrence.^3,4^ We suggest this as the method of choice for the concomitant treatment of cholelithiasis and ventral hernia.
